# Bedside Testing for Chronic Pelvic Pain: Discriminating Visceral from Somatic Pain

**DOI:** 10.1155/2011/692102

**Published:** 2011-11-03

**Authors:** John Jarrell, Maria Adele Giamberardino, Magali Robert, Maryam Nasr-Esfahani

**Affiliations:** ^1^Calgary Chronic Pain Centre and Department of Obstetrics and Gynecology, University of Calgary, 1403 29th Street NW, Calgary, AB, Canada T2N 2T9; ^2^Pathophysiology of Pain Laboratory, Department of Medicine and Science of Aging, “G. D'Annunzio” University of Chieti, Chieti, Italy

## Abstract

*Objectives*. This study was done to evaluate three bedside tests in discriminating visceral pain from somatic pain among women with chronic pelvic pain. *Study Design*. The study was an exploratory cross-sectional evaluation of 81 women with chronic pelvic pain of 6 or more months' duration. Tests included abdominal cutaneous allodynia (aCA), perineal cutaneous allodynia (pCA), abdominal and perineal myofascial trigger points (aMFTP) and (pMFTP), and reduced pain thresholds (RPTs). *Results*. Eighty-one women were recruited, and all women provided informed consent. There were 62 women with apparent visceral pain and 19 with apparent somatic sources of pain. The positive predictive values for pelvic visceral disease were aCA-93%, pCA-91%, aMFTP-93%, pMFTP-81%, and RPT-79%. The likelihood ratio (+) and 95% C.I. for the detection of visceral sources of pain were aCA-4.19 (1.46, 12.0), pCA-2.91 (1.19, 7.11), aMTRP-4.19 (1.46, 12.0), pMFTP-1.35 (0.86, 2.13), and RPT-1.14 (0.85, 1.52), respectively. *Conclusions*. Tests of cutaneous allodynia, myofascial trigger points, and reduced pain thresholds are easily applied and well tolerated. The tests for cutaneous allodynia appear to have the greatest likelihood of identifying a visceral source of pain compared to somatic sources of pain.

## 1. Introduction

Chronic pelvic pain is a complex disorder that is poorly understood. Chronic pelvic pain is defined as pain of six or more months' duration that is situated in the abdomen, groin or lower back [1]. The use of diagnostic imaging techniques such as ultrasound, CT scanning, and magnetic resonance techniques has been helpful for many gynecological conditions, but commonly the tests are normal despite the presence of severe chronic pain [2, 3]. Women suffering from chronic pelvic pain are often personally challenged because of the absence of evidence to support their complaints of severe pain [4, 5]. Recognized causes of chronic pelvic pain include visceral conditions such as endometriosis, pelvic inflammatory disease, and pelvic adhesions and somatic conditions such as lower genital tract surgery or vehicular trauma to the pelvis [6, 7]. In many cases the differentiation between visceral and somatic reasons is obvious, but in some, the distinction is not evident. This can lead to situations where investigations and surgery are repeated and in some cases extensively. 

Pain arising from the pelvic organs of the uterus, fallopian tubes, ovaries, ureter, kidney pelvis, bladder, and rectum is defined as visceral in nature. Such pain is due to the presence of visceral nociceptors present in the various organs of the female pelvis [8]. The origins of understanding of the physiology extend back to a significant period of time [9]. The concepts of referred visceral pain to a specific cutaneous location and that an irritable focus in certain tissues could be responsible for such localization extend to many years [10, 11]. Notably, stimulation of the ureter or the pelvis of the kidney was found to cause a contraction of the muscles of the abdominal wall on the stimulated side and remain contracted for a period of time [12]. Stimulation of visceral tissues causes a viscerosomatic reflex, and there is evidence this can be mediated from sympathetic efferent activity as the local pain of trigger points in the muscle is not only reduced by the administration of local anesthetic into the trigger point but can be reduced as well as by a sympathetic blockade [13, 14]. Visceral pain was also found to reduce cutaneous pain thresholds to thermal stimuli in the referred area [15].

The complexity of pain in the female reproductive system, particularly the relationship of the referral of pain through viscerosomatic processes, has recently been reviewed by Giamberardino [16]. As the visceral afferents are greatly outnumbered by somatic afferents, there is considerable merging of signals which makes the specificity of organ of origin a complex message for the central nervous system. This means that pain originating from pelvic organs may not be identified with accuracy [17, 18]. In studies of biliary disease, the presence of visceral afferents became apparent through transmission of pain from the gallbladder to the spinal cord which, when severe or repetitive, produce changes in the referred dermatomes [19]. Under these circumstances, changes occurred in the right-upper quadrant including cutaneous allodynia, muscle tenderness, and reduced pain thresholds in the subcutaneous and muscular tissues of the right upper quadrant [19]. There was a direct positive correlation between the number of colicky episodes and the degree of pain threshold reduction. The current study of the interaction of visceral disease and pelvic pain is based on these important previous investigations, particularly those evaluating biliary disease. 

The relationship of visceral disease to areas of muscle tenderness has a long history [10, 11, 20]. Extrapelvic examples of the development of myofascial trigger points from visceral disease include biliary, cardiac, and renal causes [21–23]. More specific references to the pelvic diseases include endometriosis and interstitial cystitis [23, 24]. 

The specific research question was an exploratory evaluation of the ability of simple bedside tests of cutaneous allodynia, myofascial pain, and reduced pain thresholds to differentiate women with visceral and somatic conditions associated with their chronic pelvic pain [25]. 

## 2. Methods

This was a cross-sectional study that studied the clinical assessment of women with three diagnostic tests. Eighty-one women referred by a family physician or a gynecologist for complaints of chronic pelvic pain were included in the study. The date of entry was the date of approval of ethics application at The University of Calgary which was 13 November, 2009. The date of completion was April 8, 2011. Women were approached to enter the study during a clinical consultation for the management of chronic pain. All women approached agreed to the study with the exception of one who declined for no given reason. The operational definition of visceral disease as a cause of the woman's chronic pelvic pain was pain that clinically appeared to be originating from visceral tissues. This was based on the clinical history, physical examination, referral information, and available documentation from the health records of the Calgary Health Region of Alberta Health Services. Women who were identified as having somatic pain did not have a history of visceral disease but did have prior lower genital tract surgery, lower genital tract obstetrical trauma, or musculoskeletal disorders of the pelvic bones from prior motor vehicle accidents. For this study the individual therapies for pain were not collected.

The clinical testing was undertaken contemporaneously with the pain evaluation and collection of all relevant medical history. The testing was done by a single unblinded observer. All women complained of chronic pelvic pain for more than six months. Women were selected on the basis of convenience given the availability of clinic time. The study was approved by the ethics committee of the University of Calgary. All women provided informed consent for the project.

Testing for cutaneous allodynia involved the use of a cotton-tipped culture stick as previously demonstrated [26] and validated [27]. The culture stick is drawn down from the upper abdomen into the area identified as painful by the woman. In the presence of cutaneous allodynia there is a sharply demarcated area in which this sensation goes from nonpainful to a painful sensation. The area can be variable in size, from dime-sized areas on one or both sides of the lower quadrants to broad expanses of the lower abdomen. The usual location is in the region of the dermatomes of T12-L1 located centrally in the abdomen. The same approach is undertaken on the perineum by drawing the cotton-tipped culture stick across the buttocks in a horizontal fashion to identify mainly the S3 dermatome. Preliminary studies of the validity have demonstrated blinded interrater reliability of 98%.

Within the areas of cutaneous allodynia, one can appreciate increased muscle tone and myofascial trigger points [24, 25]. The examination for myofascial trigger points has been validated [28]. These are confirmed by an examination of the abdominal wall and perineum within the area of cutaneous allodynia in which a small nodule can be palpated. The patient often will direct the examiner in detecting the painful area. When this nodule is pressed, it causes severe pain with referral of pain into the back, legs, chest, and pelvis [29]. The sensation of the pain has commonly the same characteristic as the chronic pain being experienced. When the pressure is released, the pain resolves. The areas tested for this study included the right- and left-upper abdominal and the right- and left-lower abdominal quadrants. In almost all cases, the myofascial trigger points were identified in the right- and/or left-lower quadrants near the junction of the *external oblique* and *rectus abdominus *muscles. As many women with chronic pelvic pain have difficulty associated with sexual relations, owing to myofascial dysfunction on the perineum, we also included testing of the presence of a trigger point on the perineal body.

Pain threshold evaluation involved the use of the Von Frey electroanesthesiometer (IITC Life Science). This test has been validated for the assessment of pain [30]. It was initially applied to the deltoid muscle as a reference point or internal control. Pressure of 100 g that did not produce pain was taken as a negative test on the deltoid and other areas. Measurements of pain threshold were then taken in the right-upper and right-lower abdominal quadrants and the left-upper and left-lower abdominal quadrants and the perineal body which is located on the perineum just distal to the hymen on the posterior fourchette. On the perineum, the algometer was applied to the affected muscle by applying the instrument to a sterile culture stick. In all testing the pressure was gradually applied to the affected area until the woman identified a painful sensation or until a maximum of 100 g pressure was obtained. Pain thresholds lower than 100 g were identified as demonstrating reduced pain thresholds. The measurement was then calculated as a percentage of the deltoid measurement.

The presence or absence of cutaneous allodynia, myofascial dysfunction, and reduced pain thresholds was evaluated in relation to the clinical diagnosis to determine the sensitivity, specificity, positive predictive values, negative predictive, likelihood ratios, and odds ratios of the tests. Categorical results were evaluated with contingency methods and non- normally distributed variables were compared using the Mann Whitney *U* test. Statistical analysis was done using SPSS. Diagnostic test properties were evaluated using tests for sensitivity, specificity, positive predictive value (PPV), negative predictive value (NPV), likelihood ratio, and odds ratio with 95% confidence intervals (95% C.I.) [31].

## 3. Results

A total of 81 women who were complaining of pelvic pain for at least six months were included in the study. Of the 81 women, 62 were identified as having prior or current visceral disease and 19 were identified as having somatic causes of pain. Of the women with visceral pain, the following conditions were identified: endometriosis-36; pelvic inflammatory disease-2; adhesions-5; interstitial cystitis-1; dysmenorrhea-11; ovarian cyst removal-4, fibroid-2; tubal ligation-1. The 19 women with somatic causes of pain had clinical histories indicating trauma to the lower genital tract or pelvis from surgery or motor vehicle accidents. Four women were identified as having both visceral and somatic causes of pain. 

The mean age overall of the women was 33.9 ± 1.2 (SEM) years. They had previous gravidity status of 1.06 ± 0.16 and parity of 0.82 ± 0.13. The mean duration of pain was 4.3 ± 0.45 years. Women with visceral pain were younger (*P* < 0.01), had fewer pregnancies (*P* < 0.001), and reported a longer duration of pain (*P* < 0.01) when compared to women with somatic pain (Table 1). Women with visceral disease had a greater number of prior laparoscopies (*P* < 0.001) but similar number of laparotomies when compared to women with somatic pain (Table 1). 

The diagnostic test results are presented in Table 2. Abdominal and perineal cutaneous allodynia and abdominal myofascial trigger points were found to significantly discriminate visceral from somatic sources of pain (*P* < 0.001). Perineal myofascial trigger points and reduced pain thresholds did not discriminate visceral from somatic sources of pain. The evaluation of likelihood and odds ratios in relation to the use of these tests to detect a visceral source of pain is presented in Table 3. The likelihood and the odds ratios of a positive finding of abdominal and perineal cutaneous allodynia and abdominal trigger points significantly indicated a positive identification of a visceral source of pain compared to a somatic source of pain (*P* < 0.001) (Table 3). 

A comparison of the numeric reduction in pain thresholds identified using the Von Frey electroanesthesiometer demonstrated significantly lower pain thresholds in the right- and left-lower quadrants of women with visceral pain compared to women with somatic pain (*P* < 0.05) (Table 4). There were no differences in mean pain thresholds in the deltoid region, upper abdominal quadrants, or perineum.

## 4. Comment

This was a preliminary evaluation of three simple tests that can be used at the bedside for the evaluation of women with chronic pelvic pain. The tests are not unique and are based on historic and contemporary assessments of visceral disease [2–9]. The discriminating factor was the clinical assessment in the determination of allocation to either visceral or somatic sources of pain. 

Under these circumstances, women with an apparent visceral source of pain were younger, had fewer pregnancies and a longer duration of pain, and more frequently demonstrated abdominal and perineal cutaneous allodynia and abdominal trigger points than women with an apparent somatic source of pain. Although there was a similar trend for reduced pain thresholds, these categorical differences did not attain statistical significance. However, the actual measures of pain threshold reduction were greater among women with visceral disease in the right- and left-lower quadrants. The actual mechanism(s) associated with these changes is not known conclusively but likely relates to the phenomenon of central sensitization in the dorsal horns of the spinal cord [32–34]. 

These findings are in part consistent with the validated model of biliary disease of Giamberardino et al. and extend our understanding of visceral afferents to the problems of chronic pelvic pain [35, 36]. Although the biliary studies were able to demonstrate a relationship of the number of colicky episodes, in this study there was no relationship noted between duration of pain and the presence and severity of pain threshold reduction [37]. The lack of a reduction in pain thresholds in this study possibly reflects the Von Frey electroanesthesiometer is less sensitive than the electrical stimulation methods of Giamberardino's study. Alternatively, duration of pain may not be a good surrogate for severity.

There are limitations to this preliminary evaluation. It is based on the best available clinical judgment in relation to preexisting and current conditions. As the tests were undertaken by the clinician doing the assessments, there is a possibility of bias in the interpretation of the test findings. A study is now underway to explore these findings prior to and following operative laparoscopy so that the actual assessment of visceral disease may be concurrent with test evaluation and not historical in nature. Future studies will permit test blinding in relation to diagnostic allocation. Preliminary findings of a blinded interrater reliability study evaluating 50 women with and without pelvic pain indicate 98% agreement in the testing of cutaneous allodynia. 

In this study an allocation to apparent visceral source of pain was based on available documented history and operative notes. Women with apparent somatic sources of pain included those with lower genital tract pelvic trauma. A larger sample size will be required to explore the value of these tests in relation to specific causes of visceral conditions such as endometriosis and various somatic conditions. A longitudinal study will be required to evaluate the developmental patterns involved. In future, the potential relationship of the findings to specific therapeutics will be an important consideration.

The tests are simple to perform, based upon validated methods, and do not require sophisticated equipment. The tests are readily accepted by women because of their simplicity and potential ability to provide an acknowledgement of their pelvic pain. With a greater sample size and a prospective evaluation, the relevance to the clinical diagnosis and the relationship of test results to the longitudinal pattern of disease over time may become clearer.

## Figures and Tables

**Table 1:** Demographics of women with chronic pelvic pain.

Diagnosis		Age*	Gravida**	Para**	Duration*	Laparoscopy**	Laparotomy
Visceral	*N*	62	60	60	62	61	59
	Mean	32.3	0.8	0.6	4.8	1.7	0.4
	Median	30.5	0.0	0.0	3.0	1.0	0.0
	Std. error of mean	1.2	0.2	0.1	0.6	0.3	0.1
	Std. deviation	9.4	1.3	1.0	4.3	2.7	0.7

Somatic	*N*	19	19	19	19	16	16
	Mean	39.4	2.0	1.6	3.0	0.3	0.2
	Median	33.0	2.0	2.0	2.0	0.0	0.0
	Std. error of mean	2.9	0.3	0.3	0.6	0.2	0.1
	Std. deviation	12.4	1.5	1.3	2.6	0.8	0.4

*P < 0.01; **P < 0.001.

**Table 2:** Sensitivity, specificity, positive and negative predictive values of cutaneous allodynia, myofascial trigger points, and reduced pain thresholds among women with visceral and somatic pain.

		Visceral	Somatic		
Abdominal cutaneous allodynia	Yes	41	3	Sensitivity	0.66
				Specificity	0.84
	No	21	16	PPV	0.93
				NPV	0.43
				*P* < 0.001	

Perineal cutaneous allodynia	Yes	38	4	Sensitivity	0.61
				Specificity	0.79
	No	24	15	PPV	0.91
				NPV	0.38
				*P* = 0.001	

Abdominal trigger points	Yes	41	3	Sensitivity	0.66
				Specificity	0.84
	No	21	16	PPV	0.93
				NPV	0.43
				*P* < 0.001	

Perineal trigger points	Yes	44	10	Sensitivity	0.70
				Specificity	0.47
	No	18	9	PPV	0.81
				NPV	0.33
				NS	

Reduced pain thresholds	Yes	52	14	Sensitivity	0.83
				Specificity	0.26
	No	10	5	PPV	0.79
				NPV	0.33
				NS	

**Table 3:** Likelihood ratios and odds ratios with 95% confidence intervals in the detection of visceral sources of pain with tests of cutaneous allodynia, myofascial trigger points, and reduced pain thresholds.

	LR+	95% C.I.	OR	95% C.I.
Abdominal cutaneous allodynia	4.19	1.46, 12.0	10.41	2.72, 39.79

Perineal cutaneous allodynia	2.91	1.19, 7.11	5.93	1.76, 20.02

Abdominal trigger points	4.19	1.46, 12.0	10.41	2.72, 39.79

Perineal trigger points	1.35	0.86, 2.13	2.2	0.77, 6.13

Reduced pain thresholds	1.14	0.85, 1.52	1.85	0.55, 6.30

LR+: likelihood of a positive test.

OR: odds ratio of a positive test.

95% C.I.: 95% confidence interval.

**Table 4:** Mean pain thresholds in grams by region of the abdomen among women with visceral and somatic causes of pelvic pain.

	Diagnosis	*N*	Mean	Std. deviation	Std. error mean
Deltoid	Visceral	62	100.0		
	Somatic	19	100.0		

RUQ	Visceral	62	98.2	8.6	1.1
	Somatic	19	100.0		

LUQ	Visceral	62	98.6	8.3	1.1
	Somatic	19	100.0		

RLQ	Visceral	62	75.8*	29.1	3.8
	Somatic	19	91.5*	21.4	4.9

LLQ	Visceral	62	78.2*	27.2	3.5
	Somatic	19	93.3*	20.0	4.6

Perineum	Visceral	62	69.9	25.6	3.2
	Somatic	19	73.1	22.5	5.2

*P < 0.05. RUQ: right-upper quadrant of the abdomen. LUQ: left-upper quadrant of the abdomen. RLQ: right-lower quadrant of the abdomen. LLQ: left-lower quadrant of the abdomen.
